# AirDNA sampler: An efficient and simple device enabling high-yield, high-quality airborne environment DNA for metagenomic applications

**DOI:** 10.1371/journal.pone.0287567

**Published:** 2023-06-29

**Authors:** Piyanun Harnpicharnchai, Panyapon Pumkaeo, Paopit Siriarchawatana, Somsak Likhitrattanapisal, Sermsiri Mayteeworakoon, Lily Ingsrisawang, Worawongsin Boonsin, Lily Eurwilaichitr, Supawadee Ingsriswang

**Affiliations:** 1 National Center for Genetic Engineering and Biotechnology, National Science and Technology Development Agency, Khlong Luang, Pathum Thani, Thailand; 2 Department of Statistics, Faculty of Science, Kasetsart University, Chatuchak, Bangkok, Thailand; 3 National Energy Technology Center, National Science and Technology Development Agency, Khlong Luang, Pathum Thani, Thailand; University of Hyogo, JAPAN

## Abstract

Analyzing temporal and spatial distributions of airborne particles of biological origins is vital for the assessment and monitoring of air quality, especially with regard to public health, environmental ecology, and atmospheric chemistry. However, the analysis is frequently impeded by the low levels of biomass in the air, especially with metagenomic DNA analysis to explore diversity and composition of living organisms and their components in the air. To obtain sufficient amounts of metagenomic DNA from bioaerosols, researchers usually need a long sampling time with an expensive high-volume air sampler. This work shows the utilization of an air sampling device containing an economical, high-volume portable ventilation fan in combination with customized multi-sheet filter holders to effectively obtain high yields of genomic DNA in a relatively short time. The device, named ‘AirDNA’ sampler, performed better than other commercial air samplers, including MD8 Airport and Coriolis compact air samplers. Using the AirDNA sampler, an average DNA yield of 40.49 ng (12.47–23.24 ng at 95% CI) was obtained in only 1 hour of air sampling with a 0.85 probability of obtaining ≥10 ng of genomic DNA. The genomic DNA obtained by the AirDNA system is of suitable quantity and quality to be further used for amplicon metabarcoding sequencing of 16S, 18S, and cytochrome c oxidase I (COI) regions, indicating that it can be used to detect various prokaryotes and eukaryotes. Our results showed the effectiveness of our AirDNA sampling apparatus with a simple setup and affordable devices to obtain metagenomic DNA for short-term or long-term spatiotemporal analysis. The technique is well suited for monitoring air in built environments, especially monitoring bioaerosols for health purposes and for fine-scale spatiotemporal environmental studies.

## Introduction

Biological airborne particles, also known as bioaerosols, are collective terms for diverse airborne materials mostly derived from living and dead organisms including bacterial cells, fungal spores and hyphae, viruses, and algae, as well as pollens and cellular fragments derived from plants and animals [1, 2]. These particles are largely aerosolized from a variety of environments of both natural and anthropogenic sources [3, 4] and they play vital roles in influencing particularly the public health, reproduction and spread of various organisms, climate, and precipitation cycle of the earth [2]. For example, bioaerosols have been known to cause certain infections, allergies, toxic reactions, and other respiratory and neurological disorders in human beings and other organisms, impacting health on local and global scales [4]. Thus, it is important to assess and control airborne microbes and particles for air-quality monitoring.

Investigation of microbiome present in the air has traditionally be the main focus of bioaerosol studies that include how various airborne microorganisms and their interaction with one another can influence the health of human beings and the environment. Conventionally, the detection of microorganisms presents in the air relied on growing the collected microorganisms on culturing media (culture-based approach). However, it has been proposed that only a small fraction of microorganisms from environments can be readily grown in standard laboratory conditions [5]. Therefore, metagenomic technique (culture-independent approach) with DNA-based taxonomic identification is now regularly used to evaluate microbial diversity in the air samples as well as interactions among different microbes and their surrounding environment. Most culture-independent bioaerosol studies usually employ PCR to amplify targeted genes or regions such as the 16S rRNA gene (for prokaryotes) as well as the 18S rRNA gene or the internal transcribed spacer (ITS) region (for eukaryotes) followed by amplicon sequencing [6, 7]. Additionally, in another DNA-based approach, shotgun metagenomic sequencing allows for unbiased, whole-genome investigation of microorganisms in the bioaerosols. The progress made with molecular, culture-independent techniques has great potential for novel discoveries with applications related to health, climate, agriculture, and environment [8–12]. Recently, in addition to detection of microorganisms, it has been shown that metagenomic approach has allowed detection of DNA of plants, invertebrate animals, and vertebrate animals from bioaerosols as a comprehensive mean of diversity analysis in a particular environment without the need for expertise in morphology identification associated with traditional methods [13–16]. For example, due to high environmental specificity, detection of Hemiptera (true bugs) DNA in bioaerosols had been proposed for utilization as a bio-indicator of various environmental parameters, such as habitat structure [17]. An efficient method of bioaerosol collection will thus be of benefit for survey and characterization of organisms, especially with regard to invasive or rare organisms or organisms with health impact to human beings.

Even though there is an increasing interest in bioaerosol metagenomics to enhance our understanding of the diversity and dynamics of living organisms in the environment, the progress of metagenomics with DNA collected from bioaerosols has still been lagging behind those of metagenomics of other environments (e.g. soil, marine, freshwater, and human gut) due to several challenges. First, components of microorganisms and macroorganisms in the air are generally present in lower concentrations than in other environments. These can lead to detection limits and sensitivity problems in subsequent analysis [18, 19]. Especially, bacteria and fungi have been found at low concentrations in the air, with up to 10^6^ and 10^7^ cells/m^3^, respectively [11, 20, 21]. The low concentrations of biomass in the air often lead to difficulties in studying diverse organisms from the air samples, especially as a downstream molecular analysis with genomic DNA usually requires an adequate amount of DNA to be subjected to the analysis. For example, for metabarcoding analysis to identify diverse organisms in the air samples, typically 10–20 ng of genomic DNA template is needed [22, 23] and shotgun metagenomic sequencing generally requires relatively high quality and quantity of DNA [24, 25]. Collecting enough airborne environmental DNA, usually from thousands of liters of air, for metagenomic analysis is thus often a challenge [24, 26, 27]. Relating to the low biomass present in the air, efficient retrieval of airborne microorganisms with appropriate air samplers and optimization of air sampling protocol and/or DNA extraction methods are often needed. Another challenge is the analysis relating to DNA sequencing and bioinformatics. Generally, metagenomic data can be overrepresented and biased toward the higher abundant species compared to the low-abundant species [28]. This problem can be somewhat mitigated by deep sequencing of DNA and by increasing the depth of coverage to ensure that low abundant microorganisms are represented at least once [8]. However, the deep sequencing requires high quantities of starting genetic material, which is a challenge for bioaerosol samples as stated above [8]. The third challenge in biological aerosol study is that bioaerosol compositions in the air can vary greatly and they constantly change due to both natural and anthropic factors [29–31]. This often make direct comparisons among samples and comparisons across different studies difficult. Many sampling repetitions may have to be done to mitigate the effects of these variations. However, if it is desirable to study these temporal and spatial heterogeneities, short-term, rather than long-term, sampling period will be more conducive to the analysis.

It can be seen that an effective sampling protocol can aid in the metagenomic analysis of biological aerosols. In particular, improving the collection efficiency of air sampling devices can ensure sufficient amounts of airborne cells and bioparticles are captured. Currently, with regard to air sampling devices, there is no consensus standardized protocol for collecting bioaerosol for molecular studies. Air sampling can be achieved with different methods including filtration, impaction, impingement, or cyclone technology [32–37]. Filtration usually captures bioaerosols by passing air (with air sampling pumps) through filter membranes. Impaction involves impacting the samples onto collection surfaces such as agar or gelatin plates after collecting them through nozzles. Impingement passes air through air nozzles and bioaerosols are captured in a liquid collection medium as the air passes into the liquid. Cyclones use centrifugal forces to capture bioaerosols on the surface of the passage. It was reported that the filter-based method usually recovers comparable or more genomic DNA (represented by higher gene copies or DNA yields) obtained than impaction and liquid impingement [24, 38], which may be due to generally higher air flow rate of filter-based air sampler or the possible loss of some microbial cells during centrifugation of impinger’s collection liquid. In addition, some collection liquid in the impinger may evaporate and cause the loss of microbial cells. However, in most cases, impingers and impactors may give higher numbers of viable cells than filter-based air samplers, presumably because the samples collected on filter membranes are most likely to be subjected to a higher degree of desiccation, especially after prolonged sampling time [24, 38]. Thus, the air sampler should be chosen according to applications and purposes of the work.

The present study aims to increase yields of genomic DNA derived from prokaryotes and eukaryotes present in bioaerosols by devising strategies to enhance the performance of bioaerosol collection. In the case of our air sampling, improvement of bioaerosol collection with a filter-based air sampler with simple, affordable portable ventilation fan and customized multi-sheet filter holders were investigated for a convenient and effective method to obtain high DNA yields from bioaerosols that can be further used for accurate and reliable molecular analysis including metabarcoding and metagenomic sequencing.

## Materials and methods

### Bioaerosol sample collections

Unless stated otherwise, the samples were collected at the same location in the laboratory at National Science and Development Agency (NSTDA) as a representative of the low-biomass indoor environment (Sampling A, B, and C in Table 1). The laboratory (rubber tile flooring, concrete ceiling) was equipped with a ventilation system and was approximately 6.45 х 5.1 х 2.9 m with an approximate volume of 95.4 m^3^. Samples were collected from the middle of the room to avoid disturbance from the ventilation and door. In addition, to compare DNA yields obtained from two air samplers (MD8 Airport and AirDNA samplers, see also below) in another location, air samples were collected outdoor on the ground behind the laboratory building (Sampling D in Table 1). For the assessment of air quality variable conditions that were conducive for obtaining microbial DNA from bioaerosol samplings (Sampling E in Table 1), the air samplings and air variables were conducted at the hallway next to the laboratory. The hallway was approximately 55.5 х 2.1 х 2.9 m with an approximate volume of 337.995 m^3^.

**Table 1:** Sampling time and number of samples in this study.

Sampling	Sampling Dates	Number of samples	Purpose
A	June 2021	6 samples obtained with MD8 Airport air sampler and 6 samples obtained with Coriolis air sampler (Data are provided in S2 Table, sheet A, in the S2 File)	Comparison of DNA yields from MD8 Airport and Coriolis air samplers
B	June 2021 –September 2021	41 samples obtained with MD8 Airport sampler used as training set for simulation-based estimation of the minimal number of filter sheets (Data are provided in S2 Table, sheet B, in the S2 File)	Simulation-based estimation of the minimal number of filter sheets required to obtain 10 ng of DNA by the MD8 Airport air sampler
C	September 2021 –February 2022	48 samples obtained with AirDNA sampler and 48 samples obtained with MD8 Airport air sampler (Data are provided in S2 Table, sheet C, in the S2 File)	Assessment of DNA yield performances (probability density) and PCR performance of the MD8 Airport and AirDNA air samplers (indoor location in the laboratory) (see also below for description of the AirDNA sampler)
D	July 2022	16 samples obtained with AirDNA sampler and 16 samples obtained with MD8 Airport air sampler (Data are provided in S2 Table, sheet D, in the S2 File)	Assessment of DNA yield performances (probability density) of the MD8 Airport and AirDNA air samplers (outdoor location outside the laboratory building)
E	December 2021 –May 2022	66 samples obtained with AirDNA sampler (Data are provided in S2 Table, sheet E, in the S2 File)	Assessment of air quality variable conditions that are conductive for obtaining microbial DNA from AirDNA Sampler

As the duration of short-term air sampling to obtain appropriate amounts of DNA could be selected depending on factors such as human activities, air sampling flow rate, and detection limit [39, 40], 1-h sampling (hourly) duration was chosen according to the average time a person spend in that room each time and to possibly allow further investigation of effects on short-term exposure to bioaerosols or variations of microbial compositions brought into the room by human beings [41, 42]. The sampling time and numbers of samples for the hourly collections are shown in Table 1 and descriptive statistics of DNA yield for these samples are shown in S1 Table 1 in S1 File.

In the experiment to compare DNA yields obtained from filter-based technology and cyclonic technology, the MD8 Airport air sampler (Sartorius AG, Göttingen, Germany) with a flow rate of 25–125 L/min represented filter-based air sampler and the Coriolis Compact (Bertin Instruments, Bretonneux, France) with a flow rate of 50 L/min was employed with cyclone technology. Setting and handling with these air samplers were done according to the manufacturer’s manuals. The difference in DNA yields obtained by the two samplers was analyzed using Student’s t-test in R version 4.0.1 [43].

We also developed an ‘AirDNA sampler’ strategy with a combination of a portable ventilation fan (PVF) and customized multi-sheet filter holders to obtain high amounts of genomic DNA from bioaerosol (see Design of ‘AirDNA’ air sampler system for bioaerosol sampling below). The PVF was operated according to the manufacturer’s manuals.

With the MD8 Airport and AirDNA samplers, polyethersulfone (PES) membrane filters (47 mm in diameter) with 0.45 μm pore size were used throughout the study. The membranes were handled with sterile forceps. The PES membrane filter was chosen for air sampling in this study because it was reported to surpass other filters (MCE, PA, PTFE, and PVDF) significantly in quantitative and qualitative recovery of bacterial, fungal, and eukaryotic DNA [16].

During the air sampling, air quality variables were measured and recorded every 30 min in parallel with the bioaerosol collections. Particulate matters (PM_0.3_, PM_0.5_, PM_1.0_, PM_2.5_, PM_5.0_, and PM_10_), ambient temperature (AT), relative humidity (RH), dew point (DP), and wet-bulb temperature (WB) were monitored using RS-9880 Data Logging Air Quality Meter (RS Components, Bangkok, Thailand) according to the manufacturer’s recommendation.

### Design of ‘AirDNA’ air sampler system for bioaerosol sampling

**Fig 1:**
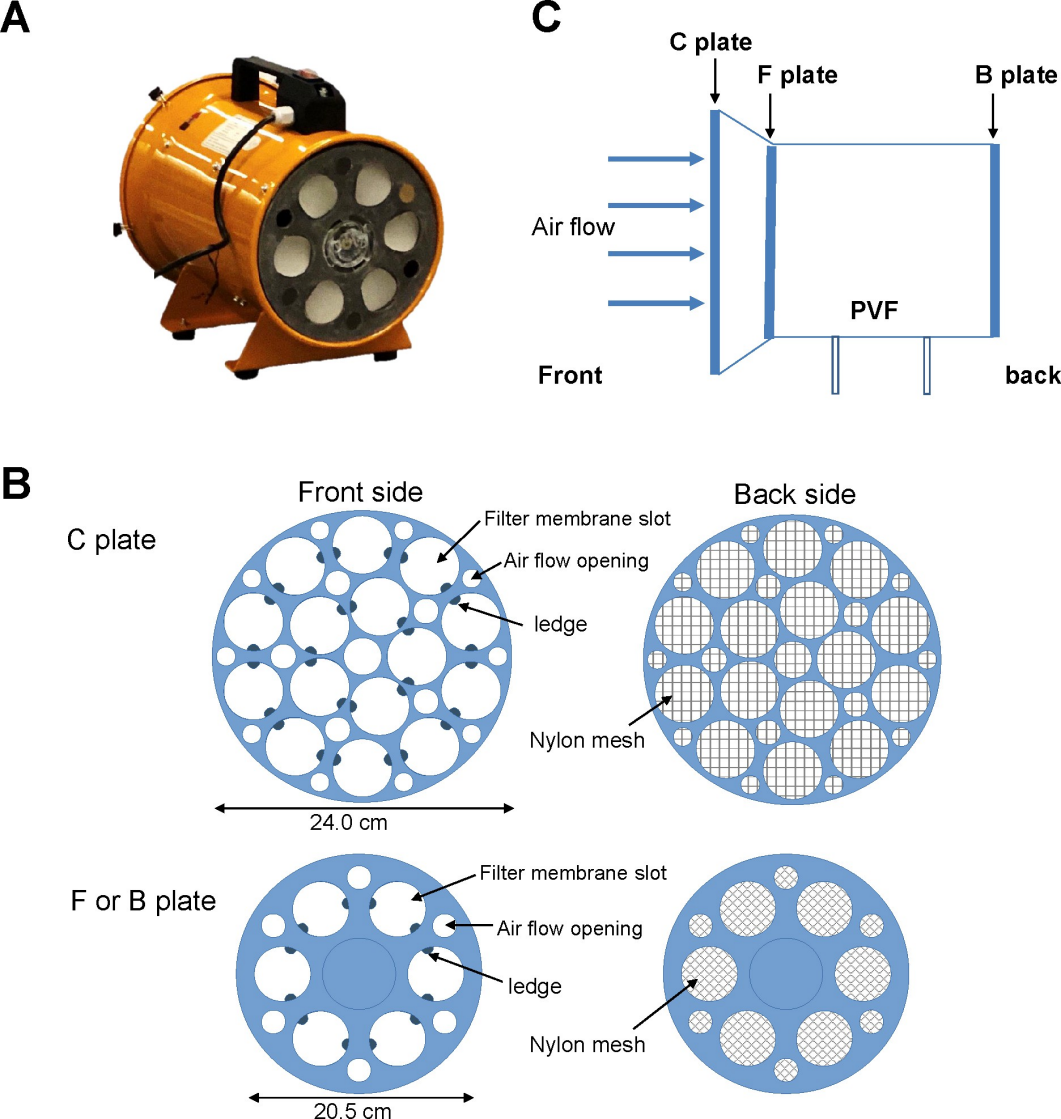
The AirDNA system used for air sampling contains portable ventilation fan (PVF) and multi-sheet filter holders. (A) The AirDNA system used in this study contained portable ventilation fan (PVF) and multi-sheet filter holders. (B) The graphical illustration showing the front view and back view of the C, F, and B multiple-sheet filter holder plates. (C) Side-view illustration showing the multiple-sheet filter holders (C plate, F plate, and B plate) mounted onto the foremost, front, and back positions onto the PVF.

In order to effectively obtain high amounts of genomic DNA derived from bioaerosols, an ‘AirDNA sampler’ system was developed. For this purpose, a standard high-volume, off-the-shelf PVF was used (Fig 1A). The PVF is a fan with a 130W motor and seven fan blades normally used for removing undesirable smoke, odors, moisture, and other pollutants in the air. Additionally, we designed a filter holder that allowed multiple sheets of filter to collect air samples simultaneously with the PVF (Fig 1B and 1C). The filter holder was a circular plastic plate designed to mount on top of the front or back casing of the portable ventilation fan. Multiple round-shaped slots, each of which has a diameter of 47 mm, functioned as slots to insert a circle-shaped filter membrane onto the holder plate. In addition, the inner rim of each of these filter membrane slots was equipped with 2 small, inwardly protruding ledges to securely accommodate the placing of filter membranes in the holder. Small holes (with diameters ranging between 1.5–3 cm) were bored through the remaining space of the holder. These holes functioned as the airflow openings to allow relatively constant airflow through the apparatus. Finally, fine nylon mesh was applied to the back side of the holder to hold filter membranes in place during the sampling.

During the collection of air samples, three multi-sheet filter holders could be mounted on the PVF in a cascade manner and used simultaneously to collect air samples. The ‘C’ plate with a diameter of 27 cm was placed on the foremost (outer front) opening of the PVF casing. The ‘F’ (20-cm-long diameter) and ‘B’ plates (20-cm-long diameter) were mounted on the immediate front (inner front) and back fan gratings, respectively. The C plate carried up to 15 filters, while each of the F and B plates could hold up to 6 sheets. Thus, the AirDNA apparatus could collect bioaerosols with up to 27 filter membranes simultaneously.

The multiple-sheet filter holder contained slots for filter membranes, small opening to allow constant air flow, ledges to securely accommodate the placing of filter membranes in the holder, and small holes for latching the filter holder onto the portable ventilation fan. At the back of the filter holder, fine nylon mesh was applied to hold filter membrane in place during the sampling.

### DNA extraction and DNA quantification

DNA extraction was conducted based on a protocol described by Crump et al. with modifications [44]. After sampling, the filter membranes were cut into small rectangular pieces (approximately 1 cm x 1 cm in size) and put into a 2-mL Eppendorf tube. Lysis buffer [0.1 M TE, 1% (w/v) SDS, and 200 μg/mL proteinase K] was added to the sample and incubated at 65°C for 2h. Then, 5M NaCl and pre-warmed 10% CTAB were added to the sample. The tube was mixed and subjected to water-bath sonication at 65°C for 30 min. The sample was vortexed horizontally for 10 min and centrifuged at 16,000 × g for 1 min. The resulting DNA-containing solution was subjected to chloroform: isoamyl alcohol [24:1 (v/v)] extraction and DNA was recovered by ethanol precipitation. Finally, to remove remaining contaminants that might inhibit downstream DNA analysis, the DNA was purified by GeneJet Gel Extraction Kit (Thermo Fisher Scientific, Massachusetts, USA.) and eluted with elution buffer supplied with the kit or dH_2_O. Negative control were conducted by DNA extraction using empty filter membrane without prior exposure to bioaerosols. DNA concentrations were quantified with a fluorometric method (Qubit dsDNA HS Assay Kit, Thermo Fisher Scientific, Massachusetts, USA.) according to the manufacturer’s instructions using 5 μL of DNA sample). The purity of the obtained DNA was assessed by the ratio of absorbance at 260 and 280 nm (A_260_/A_280_) and the ratio of absorbance at 260 and 230 nm (A_260_/A_230_) measured with UV Vis Spectroscopy.

### PCR amplification at 16S rRNA, 18S rRNA, and COI regions

Amplifications of 16S rRNA, 18S rRNA, and COI targeted regions were performed using the sequence-specific primers (Table 2). All PCR reaction was amplified using Phusion® High-Fidelity DNA Polymerase (Thermo Fisher Scientific, Massachusetts, USA.). The thermal profile of the PCR for 16S rDNA amplification was as follows: 95°C (3 min) with 34 cycles of 95°C (30 s), 55°C (30 s), 72°C (30 s), and a final extension of 72°C (5 min). The thermal profile of the PCR for 18S rRNA and COI regions was as follows: 98°C (30 s) with 30 cycles of 98°C (10 s), 50°C (30 s), 72°C (15 s), and a final extension of 72°C (10 min). Positive controls for PCR were performed using genomic DNA of *Escherichia*. *coli* (DH5α strain) as template for 16S rDNA amplification and genomic DNA of *Ogataea thermomethanolica* (TBRC 656) as template for 18S rRNA and COI amplification. The DH5α *E*. *coli* was previously purchased from Thermo Fisher Scientific (Thermo Fisher Scientific, Massachusetts, USA.) and the *O*. *thermomethanolica* (TBRC 656) was obtained from Thailand Bioresource Research Center. Negative control for PCR was performed with nanopure H_2_O instead of DNA template. The PCR products were subjected to agarose electrophoresis and visualized with FluoroDye™ DNA Fluorescent Dye (SMOBIO Technology, Taiwan, China).

**Table 2:** List of primers that used in this study.

Primer name	Sequence	Target region	Reference
314F	5′-CCTACGGGNGGCWGCAG-3′	16S rDNA	[45]
805R	5′-GACTACHVGGGTATCTAATCC-3′	16S rDNA	[45]
1391F	5’-GTACACACCGCCCGTC-3’	18S rDNA	[46]
EukBrR	5’-TGATCCTTCTGCAGGTTCACCTAC-3’	18S rDNA	[47]
BF1	5’-ACWGGWTGRACWGTNTAYCC-3’	COI	[48]
BR2	5’-TCDGGRTGNCCRAARAAYCA-3’	COI	[48]

### Statistical and computational analysis

#### Estimation of the minimal number of filter sheets for sufficient DNA yield using the MD8 Airport air sampler

In order to estimate the minimal number of filter sheets for obtaining sufficient DNA yields, simulated 100 DNA yield values were generated for each number of filter sheets from the Poisson distribution of the observed values of DNA yield concentration (in ng/sheet) with 40 degrees of freedom. The observed DNA yield values used in the simulation came from the actual yields of DNA extracted from the 41 hourly samples with the MD8 device (Sampling B in Table 1). The lower bound of one-tailed 90% confidence interval (90% CI lower bound) of those simulated values was calculated for each number of filter sheets. A linear regression line between simulated DNA yields and the number of filter sheets was plotted, and then the minimal number of filter sheets was estimated by an intersection point between the regression line and the DNA yield value = 10 ng, which should be conducive for further use in amplicon metabarcoding or shotgun metagenomic sequencing [22, 23, 49].

#### Assessment of DNA yield performance between the AirDNA and MD8 Airport air samplers

The yield performance of air sampling was evaluated using the estimated DNA yield probability density function. Mean, standard deviation, skewness, and kurtosis of DNA yields extracted from the AirDNA and MD8 samples (Sampling C and D in Table 1) were used for calculated parameters of skew-t distribution function using ‘dp2cp’ in ‘sn’ R package. The skew-t distribution function was plotted to the probability density curve using ‘geom_density’ with Gaussian kernel in the ‘ggplot2’ R package [50]. An empirical cumulative distribution function (eCDF) was employed to calculate an area under the curve (AUC) of the distribution function where DNA yields were equal to or more than 10 ng. The calculated AUCs were represented as the yield performance estimates of PVF and MD8 sampling.

#### Quantitative comparisons of PCR products using DNA obtained with the AirDNA and MD8 Airport air samplers

PCR were performed with equal volume of DNA templates, which were obtained on the same day of samplings using the AirDNA and MD8 airport air samplers. The DNA templates used were chosen from the sampling day that yielded the highest concentration of DNA obtained by the MD8 Airport sampling. In order to compare the intensities of PCR bands in gel electrophoresis experiments, digital images of gels were taken with the ChemiDoc™ MP Imaging System (Bio-Rad Laboratories, California, USA.). For each gel image, the bright PCR bands of correct size in all targeted-marker lanes were masked over by rectangles of equal sizes. The light intensity of each PCR band in the image was calculated by the ImageLab software available with the ChemiDoc™ MP Imaging System. The mean intensity values of PCR bands from the MD8 Airport and AirDNA samples were then compared using Student’s t-test.

#### Linear discriminant analysis of air quality conditions affecting DNA yields when using AirDNA sampler

In order to specify the variable importance of air variables in which the AirDNA sampling can obtain DNA yield of more than 10 ng, linear discriminant analysis (LDA) was performed to find a discriminant power of air quality properties that separates sufficient and insufficient DNA yield samples. The observed air quality properties used in this analysis came from the 66 hourly sampling with the AirDNA device (Sampling E in Table 1). Air quality properties were standardized by scaling and centering then performed linear discriminant analysis using ‘train’ in ‘caret’ R package with ‘lda’ model [51]. Variable importance was calculated using ‘varImp’ in ‘caret’ R package. The air variables included PM (PM_0.3_, PM_0.5_, PM_1.0_, PM_2.5_, PM_5.0_, and PM_10_), ambient temperature (AT), relative humidity (RH), dew point (DP), and wet-bulb temperature (WB).

## Results

### DNA yield obtained from filter-based technology and cyclonic technology

To investigate the efficacy of using air samplers with either filter-based technology or cyclonic technology to obtain genomic DNA for further molecular analysis, the performances of MD8 Airport (using filter-based technology) and Coriolis Compact (using dry cyclonic technology) air samplers with regard to DNA yield per hour of air sampling in an indoor environment were compared (Sampling A in Table 1), as both air samplers had been reported to be effective in microbial retrieval, compact, and portable (suitable for transportation to various locations of interest). Student’s t-test analysis showed that the MD8 Airport air sampler performed significantly better than the Coriolis compact, as approximately twice as much extracted DNA (0.258 ± 0.04 ng) were obtained from sampling with the MD8 Airport compared to that obtained from sampling with the Coriolis Compact (0.11 ± 0.12 ng of extracted DNA) (p-value < 0.001).

**Fig 2:**
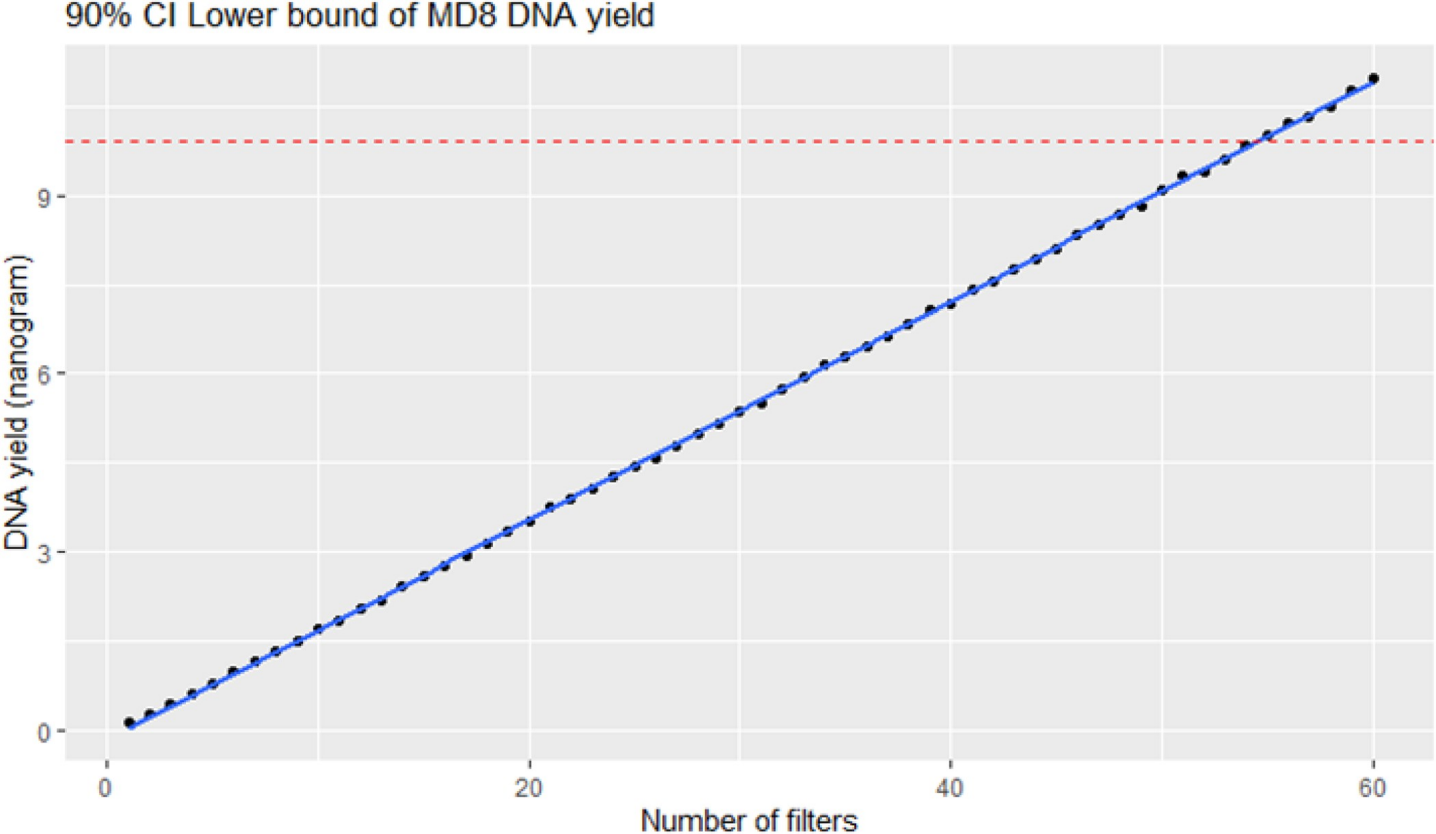
Linear regression line between simulated lower bound of DNA yields at 90% confidence interval and number of filter membranes. The estimated minimal number of filter sheets for DNA yield of 10 ng is found at an intersection point between the regression line (blue) and the DNA yield value of 10 ng (horizontal red, dashed line).

As the MD8 Airport air sampler with filter-based technology could be employed to obtain a higher amount of genomic DNA than the Coriolis Compact device, the DNA yield obtained from sampling with the MD8 Airport device was then explored further (Sampling B in Table 1). Since the MD8 Airport device employs one filter membrane at a time of each sampling, linear regression analysis was performed to estimate the minimal number of filter membranes required to obtain a target amount of genomic DNA (10 ng) with 1-h sampling time. The results in Fig 2 showed that, at 90% confidence interval, a minimum of 54 sheets of filter membranes would be required to obtain 10 ng of genomic DNA when the MD8 Airport air sampler was employed.

### Comparative analysis of DNA yields obtained with the AirDNA and MD8 Airport air samplers

From the estimation above, using the MD8 Airport air sampler with one filter membrane per hour, it would require at least the samplings of 54 h to obtain satisfactory quantity of genomic DNA. Alternatively, to reduce the time of the sampling, multiple numbers (up to 54) of the MD8 Airport device are most likely needed to be operated simultaneously. The procedure is, therefore, either time-consuming or resource-consuming. Thus, it is of advantage to devise a useful alternative to the sampling with the MD8 Airport air sampler.

It can be seen that a simple, cost-effective, high-volume portable air sampler should be constructive for collecting a higher amount of bioaerosols, particularly as sampling with multiple numbers of filter membranes may be required. To this end, the ‘AirDNA’ device was developed (see method: the Design of ‘AirDNA’ air sampler system for bioaerosol sampling and Fig 1A). Especially, the wide surface area of the air sampler allows for large or multiple filters to be used, and the high-volume feature allows for a fast collection of bioaerosols. In addition, in an attempt to efficiently enhance DNA yields, a customized filter-holder apparatus was created in the laboratory to allow applying multiple sheets of filter membranes (up to 15 membranes in our case) simultaneously with the air sampler (see Fig 1B). A higher yield of DNA may also be achieved by using a cascade of multiple filter holder plates (designated ‘C’, ‘F’, and ‘B’ plates) to allow even more filter membranes to be used during the air sampling (Fig 1C). This device can thus be employed to collect bioaerosols with 27 filter membranes at a time in accordance with the surface area and airflow rate of the sampler.

**Fig 3:**
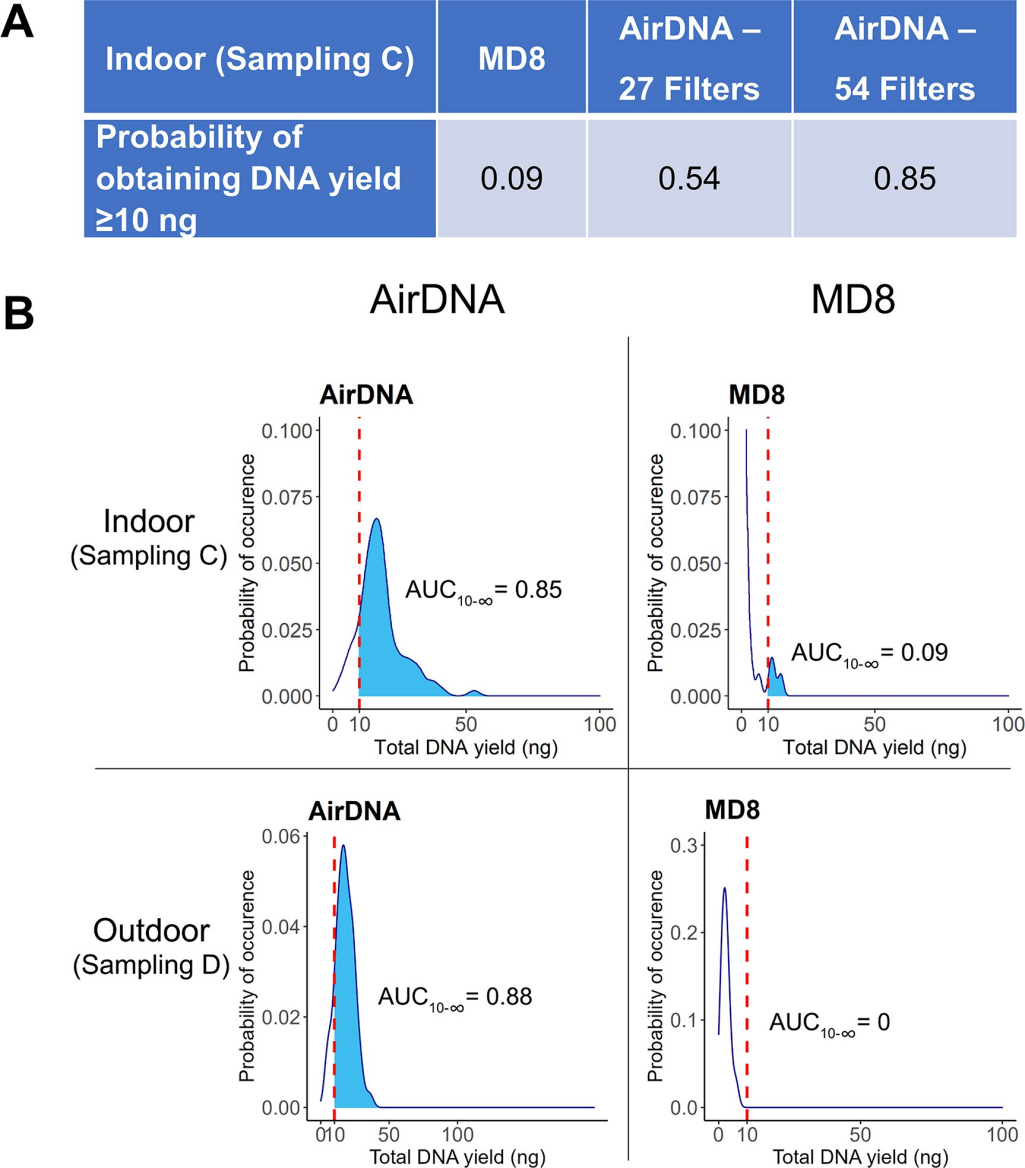
(A) Probability of obtaining DNA yield ≥10 ng in indoor location and (B) Probability density of DNA yield by the AirDNA system and MD8 Airport sampler: comparing between indoor and outdoor location. In panel A, indoor samples were used to estimate the probability. In panel B, the probability density plots were shown with both indoor and outdoor data. the Area Under the Curve (AUC) shaded in blue shows the probability of obtaining ≥10 ng of DNA.

When the performances of the AirDNA and MD8 Airport devices were compared (Sampling C in Table 1), probability density values of DNA yield revealed that using the AirDNA system allowed a significantly higher yield of DNA to be obtained than using the MD8 Airport air sampler (Fig 3). The probability of obtaining ≥10 ng of DNA yield within 1 hour using the AirDNA system with 27 sheets of filter membrane was 0.54 (Fig 3A and S1 Fig 1 in S1 File) and the average DNA yield obtained was 18.1 ng. On the other hand, the probability of obtaining ≥10 ng of DNA using the MD8 Airport air sampler in 1 h was only 0.09 (Fig 3A and 3B). Furthermore, as it was earlier estimated that collecting bioaerosols with the MD8 Airport sampler would require 54 sheets of filter membrane to get ≥10 ng of genomic DNA, we alternatively explored DNA yields resulting from using 54 sheets of filter membrane with the developed AirDNA sampler. Since the PVF is relatively low-cost and readily available off-the-shelf, simultaneous bioaerosol collection with two sets of the AirDNA system can be easily accomplished. When two units of the AirDNA sampler were simultaneously employed with a total of 54 sheets of filter membrane, the probability of obtaining ≥10 ng of DNA yield within 1 hour increased to 0.85 (Fig 3A and 3B). In this case, the average DNA yield and the yield at 95% confidence interval were 40.49 ng and 12.47–23.24 ng, respectively. Additionally, to investigate DNA yields from air samples obtained in another location, air samples were collected from an outdoor location using the MD8 Airport and the AirDNA samplers (Sampling D in Table 1). It was found that, similarly to the indoor samples, using the AirDNA sampler also resulted in higher probability to obtain the target yield of DNA than using the MD8 Airport air sampler. The probability of obtaining ≥10 ng of DNA using the AirDNA and the MD8 Airport air samplers in 1 h were 0.88 and 0, respectively (Fig 3B).

### Evaluation of the quality of the obtained DNA with PCR at targeted regions on prokaryotes and eukaryotes’ genome

**Fig 4:**
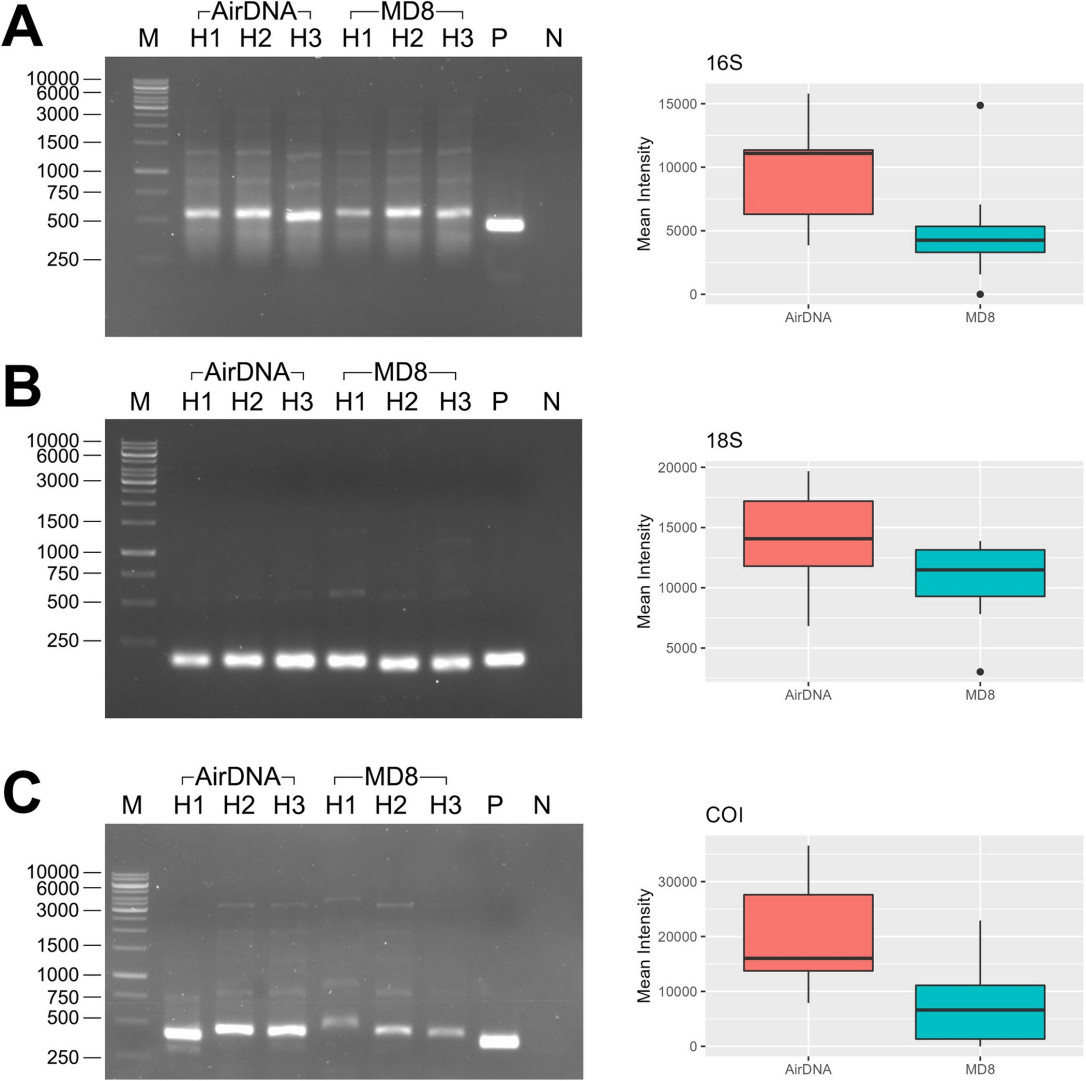
PCR products from air samples indicate (A) amplified 16S rDNA region, (B) amplified 18S rRNA region, and (C) amplified COI region. The left panel showed PCR products visualized by DNA-binding fluorescent dye after agarose gel electrophoresis. DNA lanes labeled ‘AirDNA’ indicate PCR products after using DNA obtained from the AirDNA samples as templates. DNA lanes labeled ‘MD8’ indicate PCR products after using DNA obtained from MD8 Airport samples as templates. H1, H2, and H3 denote the PCR products using DNA templates obtained from the first hour, second hour, and third hour, respectively, of samplings. Lane M indicates DNA ladder. Lane P indicates positive control for PCR products, whereas lane N indicates negative control no template) for PCR products. The right panel of Fig 4 includes box plots showing the intensity levels of PCR bands in each lane. The intensity of PCR product bands after using DNA obtained from AirDNA samples as templates are shown in orange and the intensity of PCR products after using DNA obtained from MD8 Airport samples as templates are shown in blue.

To evaluate whether the quality of DNA obtained after sampling with the AirDNA sampler system was suitable for further molecular analysis, PCR amplifications of the 16S rRNA, 18S rRNA, and COI regions were conducted. The findings demonstrate that PCR products of expected sizes could be produced (Fig 4), indicating that the DNA most likely contained appropriate quantity and quality for use in further investigation. Importantly, it also demonstrated that the obtained DNA originated from diverse organisms, including prokaryotes (mostly detected with 16S rRNA gene metabarcoding), eukaryotes especially fungi (mostly detected with 18S rRNA gene metabarcoding), and higher eukaryotes such as insects and other animals (mostly detected with COI gene metabarcoding).

The intensities of PCR products visualized after agarose gel electrophoresis were then analyzed digitally (see method: Quantitative comparisons of PCR products using DNA obtained with the AirDNA and MD8 Airport air samplers). The results revealed that when an equal volume of DNA template was used in the PCR reaction, the PCR products resulting from using DNA obtained from AirDNA samples as templates showed higher intensity levels (p-value < 0.05 for 16S and COI and p-value < 0.1 for 18S) than those using DNA obtained from MD8 Airport samples as templates (Fig 4). This reinforces that the AirDNA sampler gives superior performance than the MD8 Airport air sampler with regard to total DNA yield obtained.

### Conditions of air quality variable for obtaining a high yield of genomic DNA from the AirDNA sampler

**Fig 5:**
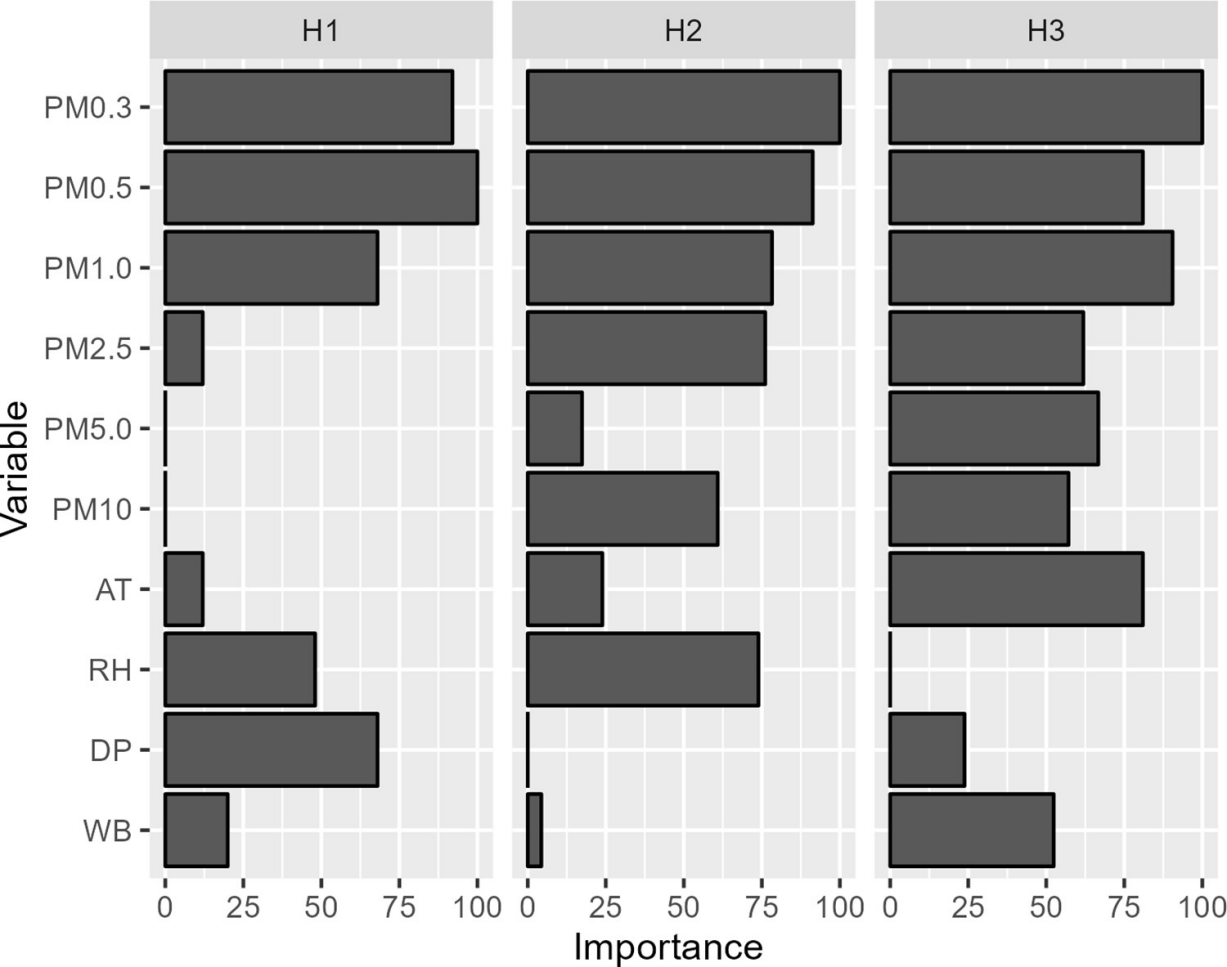
Importance levels of various air quality variables. The higher importance levels (closer to 100) suggest that the air quality variables most likely exert large effect on the DNA yields obtained. H1, H2, and H3 indicate sampling done in the first hour, second hour, and third hour, respectively.

It is beneficial to explore air quality variables that affect DNA yields. Linear discriminant analysis (LDA) with 66 hourly samples (Sampling E in Table 1) revealed that different air quality variables exerted different levels of importance on DNA yields obtained (Fig 5 and S1 Table 2 in S1 File). PM_0.3_, PM_0.5_, and PM_1.0_ were the most important variables that affect the DNA yields for all three hours (importance level > 70), while PM_2.5_, PM_5.0_, PM_10_, and AT had low importance level in first hour, but had higher importance levels in the second and the third hour. On the other hand, DP had high importance level in the first hour only. Area under the Receiver Operating Characteristic (ROC) Curve of prediction model for three hours were 0.53–0.73.

## Discussion

Given the dilute nature of biomass in the air, coupled with limitations of current DNA-based analysis with regard to detection limits and sensitivity problems, metagenomic analysis to discern spatiotemporal distributions and dynamics of the microbial community in bioaerosols, as well as biomonitoring of macroorganisms in the environment, is challenging. An efficient methodology to obtain high amount of genomic DNA will certainly help advance the metagenomic analysis of bioaerosols. It is additionally beneficial to have a reliable, methodical protocol such that researchers can collect enough biomass in a relatively short time.

In an attempt to compare the efficiency of air samplers, one of the most popular air samplers with the highest performance ranking for portable air samplers suitable for field sampling is MD8 Airport (Sartorius AG) and Coriolis Compact air samplers [52, 53]. Studies have found the MD8 air sampler to be comparable or more efficient than several other air samplers including BioSampler, Anderson viable impactor, and MAS-100 for collecting fungi and *Bacillus atrophaeus* endospores [52, 54]. Coriolis Compact air sampler is a compact high-volume dry cyclone sampler and is a new generation of the Coriolis units used for molecular analysis such as RT-qPCR detection of SARS-CoV-2 [55]. Our results indicated that the filter-based MD8 Airport air sampler can be employed to get a higher yield of genomic DNA, probably because the cyclonic device tends to be more effective in capturing large particles through centrifugal force than small and light-weight particles [56], whereas this effect is not much realized with the filter-based device. However, the DNA obtained from the MD8 Airport device is mostly insufficient to be used for metagenomic analysis.

In an effort to devise a method to obtain high DNA yield from bioaerosols for further metagenomic analysis, our ‘AirDNA’ system, using a high-volume portable ventilation fan in combination with multi-sheet filter holders, enables us to capture bioaerosols in a relatively short time of sampling. The effectiveness of AirDNA sampler in capturing bioaerosols also suggests that the device could be useful not only for characterization of airborne microorganisms, but also capturing DNA and characterization of plants and animals in the environment. This approach is straightforward and it has been used successfully to obtain a satisfactory DNA yield for further molecular analysis from the indoor environment after only 1 h of sampling. The wide surface area of the portable ventilation fan allows large or multiple sheets of filter membranes to be used simultaneously during the sampling. The PVF’s affordable and conveniently available features coupled with multiple filter holder plates make scaling up with more than 1 unit of the device easily achievable for effective bioaerosol collection. DNA obtained from multiple AirDNA samplers can also be pooled together to obtain a higher yield. In addition, the AirDNA sampler can further allow a series of filter membranes with different pore sizes to be used. That should also allow capturing different sizes of bioaerosols with different filter membranes. Even as the AirDNA sampler was first developed to collect indoor bioaerosols that usually contain low biomass, outdoor air sampling can also be simply performed with the addition of a portable battery pack, which is readily available in the local market. Other studies have also attempted to develop an aerosol collector to efficiently capture airborne microorganisms. For example, Radosevich and colleagues designed a high-volume air-sampling device modified from a dust particle collector with an 8 × 10-inch filter holder [17]. A polyester filter membrane with 1 μm pore size was used to capture bioaerosols from 1.4 × 10^6^ l of air in 24 h. From this setup, they reported a total DNA yield of <50–400 ng based on gel quantification. Our setup also yields an average of 40.49 ng DNA (and the maximum of > 300 ng DNA) in only 1 h. However, it must be cautioned that our DNA yield results cannot be directly compared to their results as there are differences in sampling location and time, DNA extraction method, and DNA quantification method. It will still be worthwhile to also compare the efficiency of the AirDNA system with other high-flow rate air samplers such as the SASS3100 air sampler [57] at the same settings even though the SASS3100 and other high-performance samplers are usually much more expensive and less conducive to transportation to different locations (due to the higher weight of SASS3100 air sampler) for spatial analysis than the PVF used in this study.

It can be seen from our results that the AirDNA system can be used to obtain genomic DNA of satisfactory quantity and quality that could be further employed for metabarcoding and shotgun metagenome sequencing. The short-term collection period should also minimize the risk of damage to biological samples and preserves the integrity of genetic materials, as prolonged air sampling duration was shown to have a negative impact on DNA recoverability [23]. In addition, the approach can most likely be further expanded for other molecular assessments including the detection of specific organisms with quantitative polymerase chain reaction (qPCR) [58] and metatranscriptomics to identify transcripts from the sample [59].

Our data of genomic DNA yields with the AirDNA system could be further used to predict significant air quality variables that affect the DNA yields. From the LDA analysis, the variable importance levels of PM_0.3_, PM_0.5_, and PM_1.0_ (with particle size ranging from 0.3–1.0 um) on DNA yields were consistently high throughout all hourly-sample groups. The effect of PM on bioaerosols have been extensively reported, as a large proportion of bioaerosols in the air is most likely attached to particles such as dust, dirt, soot, and smoke that are carried to the air from natural and human activities [60]. As smaller particulate matters usually present in higher amount than larger particles in any given time, it can be expected that the higher numbers of particles could lead to higher amounts of airborne eDNA being captured. Interestingly, the importance of PM_2.5_, PM_5.0_, and PM_10_ (with size ranging from 2.5–10 um) increased hourly. Moreover, we also found the difference in importance of hourly air quality variables, such as AT, RH, WB, and DP, on DNA yield as shown in Fig 5. These differences may imply biological and/or physical factors which can be investigated further by future studies.

Examination of the relationship between specific environmental conditions and air microbiota is important to an understanding of the impact of those environmental conditions on the microbial communities in bioaerosols. As our devised method allows the analysis of samples collected in a relatively short term, this offers the benefits of allowing a stringent and fine-resolution analysis at a short-term time scale. For example, effects of short-term exposure to bioaerosols in particular environments can be explored. In addition, more samplings (repetitions) can also be performed, which can increase the possibility of more accurate analysis even when there are usually variations in temporal and spatial distributions of bioaerosols. Most importantly, the short sampling time with the AirDNA system allows a study design that detects statistical relationships between bioaerosols and environmental conditions or air quality on multiple temporal scales, e.g. hourly, daily, weekly, monthly, and seasonally. These, in turn, can be used for modeling and predicting bioaerosol concentrations in a given environment, especially with respect to human activities. Our approach with affordable and short-time samplings can also help alleviate some of the limitations encountered with bioaerosol investigation. For example, since concentrations of indoor bioaerosols in the room can differ significantly from location to location due to human presence or airflow pattern, it is prudent to systematically conduct air samplings at multiple locations in the room many times to give reliable results. Even as the variations in bioaerosols are not of concern, multiple samples can be collected and pooled together. Our proposed air sampling with the AirDNA device is also beneficial when the number of people or the time they spend in a sampling location is limited.

## Conclusions

Our air sampling approach with the AirDNA device containing PVF and multi-sheet filter holders was successfully employed to obtain high yields of genomic DNA (40.49 ng average yield and 12.47–23.24 ng at 95% Confidence Interval) extracted from bioaerosols collected in 1 h. Statistical analysis and PCR revealed that the obtained DNA was of satisfactory quantity and quality to be further used in molecular evaluation of bioaerosols including metabarcoding and shotgun metagenome sequencing. The AirDNA system’s benefits stem from 1) its use of multiple sheets of filter membrane and high-volume air collection enabling sampling collection in a short time, 2) its simplicity and affordability along with compact size, 3) its flexibility for use in either short-term or long-term spatial and temporal analyses, and 4) its adaptability to parallel sampling using multiple units of the AirDNA device. The technique is well suited for monitoring air in built environments, especially monitoring bioaerosols for fine-scale spatiotemporal environmental studies and health purposes.

## Supporting information

S1 File Supporting figures and tables. (DOCX)

S2 File Data obtained by sampling A, B, C, D, and E. (XLSX)
