# Supplementary material for: AirDNA sampler: An efficient and simple device enabling high-yield, high-quality airborne environment DNA for metagenomic applications
Source: PLoS One. 2023 Jun 29;18(6):e0287567. doi: 10.1371/journal.pone.0287567 (PMC10309600; doi:10.1371/journal.pone.0287567)
Supplement: S1 File — (DOCX) [file pone.0287567.s001.docx]

**S1 File.** **Supporting figures and tables**.


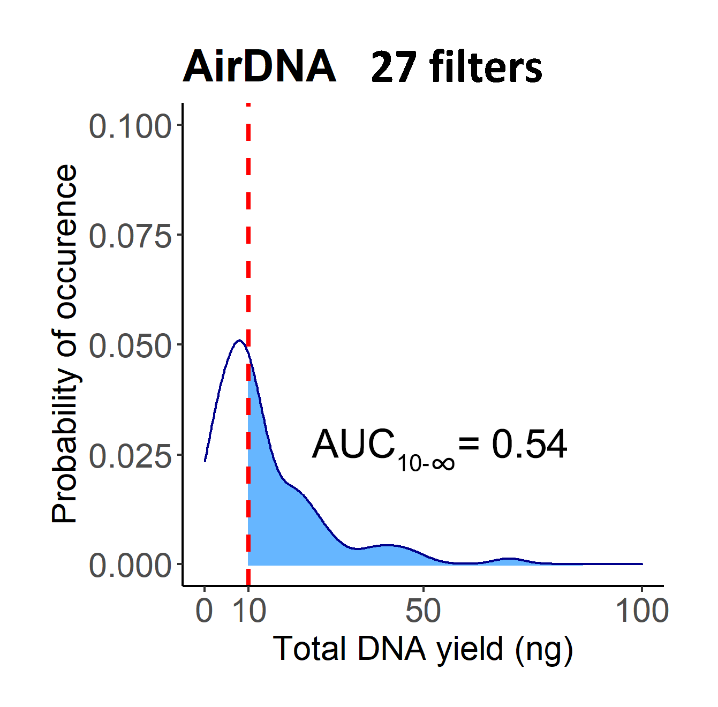


**S1 Fig 1.** **Probability density of DNA yield of air samples collected indoor by the AirDNA system using 27 filters.** The Area Under the Curve (AUC) shaded in blue shows the probability of obtaining ≥10 ng of DNA.

**S1 Table 1**. **Descriptive statistics of DNA yield data sets obtained by Sampling A, B, C, D, and E.**

| Sampling | Air samplers employed | Average DNA yield | Median DNA yield | Standard deviation | Min | Max |
| --- | --- | --- | --- | --- | --- | --- |
| A | MD8 | 0.254667 | 0.2715 | 0.042921634 | 0.188 | 0.294 |
| A | Coriolis | 0.115833 | 0.1095 | 0.016302352 | 0.1 | 0.144 |
| B | MD8 | 0.693659 | 0.24 | 1.073437833 | 0 | 5.4 |
| C | AirDNA | 42.79099 | 17.0675 | 64.88060961 | 2.45 | 337 |
| C | MD8 | 1.58774 | 0.4995 | 3.063721593 | 0 | 14.625 |
| D | AirDNA | 18.42 | 14.95 | 8.45 | 8.96 | 31.22 |
| D | MD8 | 1.94 | 1.69 | 1.75 | 0 | 6.1 |
| E | AirDNA | 63.00939 | 22.385 | 76.65665769 | 1.44 | 339.45 |

**S1 Table 2. DNA yields obtained in different hours obtained from Sampling E.**

The DNA yields obtained from Sampling E in Table 1 (66 samples) in different hours of sampling were shown. H1, H2, and H3 indicate sampling done in the first hour, second hour, and third hour, respectively.

| **Sampling Hour** | **DNA yield (ng)** | | | | |
| --- | --- | --- | --- | --- | --- |
|  | **Average** | **Standard deviation** | **50^th^ percentile** | **75^th^ percentile** | **90^th^ percentile** |
| **H1** | 69.91 | 98.96 | 24.70 | 57.62 | 229.20 |
| **H2** | 43.09 | 56.51 | 17.85 | 29.60 | 119.15 |
| **H3** | 53.95 | 70.59 | 20.05 | 50.67 | 157.48 |
